# Plasticity of Fear and Safety Neurons of the Amygdala in Response to Fear Extinction

**DOI:** 10.3389/fnbeh.2015.00354

**Published:** 2015-12-24

**Authors:** Susan Sangha

**Affiliations:** ^1^Department of Psychological Sciences, Purdue UniversityWest Lafayette, IN, USA; ^2^Ernest Gallo Clinic and Research Center, University of CaliforniaSan Francisco, Emeryville, CA, USA

**Keywords:** amygdala, fear, safety, extinction

## Abstract

Fear inhibition learning induces plasticity and remodeling of circuits within the amygdala. Most studies examine these changes in nondiscriminative fear conditioning paradigms. Using a discriminative fear, safety, and reward conditioning task, Sangha et al. (2013) have previously reported several neural microcircuits within the basal amygdala (BA) which discriminate among these cues, including a subpopulation of neurons responding selectively to a safety cue and not a fear cue. Here, the hypothesis that these “safety” neurons isolated during discriminative conditioning are biased to become fear cue responsive as a result of extinction, when fear behavior diminishes, was tested. Although 41% of “safety” neurons became fear cue responsive as a result of extinction, the data revealed that there was no bias for these neurons to become preferentially responsive during fear extinction compared to the other identified subgroups. In addition to the plasticity seen in the “safety” neurons, 44% of neurons unresponsive to either the fear cue or safety cue during discriminative conditioning became fear cue responsive during extinction. Together these emergent responses to the fear cue as a result of extinction support the hypothesis that new learning underlies extinction. In contrast, 47% of neurons responsive to the fear cue during discriminative conditioning became unresponsive to the fear cue during extinction. These findings are consistent with a suppression of neural responding mediated by inhibitory learning, or, potentially, by direct unlearning. Together, the data support extinction as an active process involving both gains and losses of responses to the fear cue and suggests the final output of the integrated BA circuit in influencing fear behavior is a balance of excitation and inhibition, and perhaps reversal of learning-induced changes.

## Introduction

Environmental cues signifying danger, safety, or reward availability can have a potent effect in emotion regulation. Accurately discriminating among these cues is important in initiating the proper emotional response in order to guide behavior. Maladaptive emotion regulation can lead to a wide-range of clinical problems, such as anxiety disorders and addiction. Since potentially rewarding and dangerous stimuli often occur simultaneously leading to opposing behaviors of approach or avoidance, respectively, reward- and fear-related circuits must interact in order to mediate these antagonistic behaviors. Approach and avoidance behaviors can also be modulated by signals that inform the organism if the environment is safe or not. The inability to discriminate among danger, safety, and reward cues can lead to generalized fear responses that are enhanced in Post-traumatic Stress Disorder (PTSD) patients (Jovanovic et al., 2012).

Behavioral therapy for maladaptive fear often involves repeated exposures to the danger cue in the absence of an aversive outcome, a procedure known as extinction. Through repeated exposures, the subject feels an increasing sense of control over the situation and fear diminishes. Safety conditioning is another method of reducing fear. During safety conditioning, a safety cue in conjunction with a danger cue signifies no aversive outcome whereas the danger cue on its own does result in an aversive outcome. Thus, extinction and safety conditioning are related but distinct phenomena. Safety cues can even act as positive reinforcers, suggesting the mechanisms of safety learning may overlap with reward learning (Christianson et al., 2012; Sangha et al., 2013).

The amygdala has been consistently implicated in processing and regulating a myriad of emotional responses (for review see Janak and Tye, 2015). The basal amygdala (BA) in particular is important for discriminating among sensory stimuli that signal multiple outcomes of a similar valence (Málková et al., 1997; Corbit and Balleine, 2005; Balleine and Killcross, 2006), and it possesses neuronal populations selective for valence (Schoenbaum et al., 1999; Paton et al., 2006; Belova et al., 2007; Shabel and Janak, 2009; Sangha et al., 2013).

Evidence suggests that fear extinction learning induces plasticity and remodeling of inhibitory circuits and synapses within the amygdala (Heldt and Ressler, 2007; Lin et al., 2009; Sangha et al., 2012), as well as decreased synaptic efficacy in the medial prefrontal cortex-BA pathway (Cho et al., 2013). Within the BA, “extinction” neurons have been reported (Herry et al., 2008). These are neurons that are unresponsive to a fear cue before extinction but become responsive to the fear cue after extinction, when fear behavior is diminished. Diminished fear behavior is also seen during safety conditioning in response to a safety cue. Using a discriminative conditioning task that allows assessment of fear, safety and reward cue learning together, Sangha et al. (2013) demonstrated significant suppression of freezing behavior in response to a compound fear+safety cue compared to the high freezing seen in response to a fear cue. In addition, this study also reported several neural microcircuits within the BA that showed a discriminative response to these cues. In particular, 24% of recorded neurons were responsive to the compound fear+safety cue but unresponsive to the fear cue when presented alone suggesting these neurons are encoding safety. Similar to these “safety” neurons, the “extinction” neurons reported by Herry et al. (2008) were also unresponsive to the fear cue before extinction training. Since safety conditioning and extinction are related phenomena, neurons classified as “safety” neurons in Sangha et al. (2013) were here examined through extinction to see if they became “extinction” neurons, similar to the neurons reported by Herry et al. (2008).

To do this, firing rates of neurons classified as discriminative, nondiscriminative or unresponsive during discriminative conditioning (DC), based on their responses to the fear cue alone and the compound fear+safety cue, were examined in response to the fear cue during extinction training and recall as fear behavior decreased. The hypothesis tested is that there is a bias for the neurons that are safety cue responsive during DC to become responsive to the fear cue as fear extinction progresses.

## Materials and methods

### Subjects

Fourteen Long Evans male rats (Harlan) weighing 350–400 g at the beginning of experiments were single housed under a 12 h light/dark cycle (lights on 07:00) and handled for 1 week before commencing experiments. All procedures were performed during the light cycle and approved by the Gallo Center Institutional Animal Care and Use Committee in accordance with the National Institute of Health guidelines. Rats had *ad libitum* access to food and water up until the third reward learning session, at which point they were restricted to 22 g of food per day for the remainder of the experiment.

### Behavioral apparatus

The experimental chambers, used in all experiments and obtained from MedAssociates, were Plexiglas boxes (32 cm length × 31 cm width × 35 cm height) encased in sound-attenuating shells. A recessed port 3 cm above the floor and located in the center of one wall was used to deliver sucrose. Two lights (28 V, 100 mA) located 12 cm from the floor on the wall opposite the port provided constant illumination. A light (28 V, 100 mA) located 33 cm above the floor on the wall opposite the port served as the 20 s continuous light cue. A high-frequency “tweeter” speaker (ENV-224BM) located 25 cm from the floor on the wall opposite the port was used to deliver the auditory cues. Footshock was delivered through a grid floor via a constant current aversive stimulator (ENV-414S). A video camera located at the top of the sound-attenuating shell recorded the rat's behavior for offline video analysis.

### Discriminative conditioning

**Figure 1:**
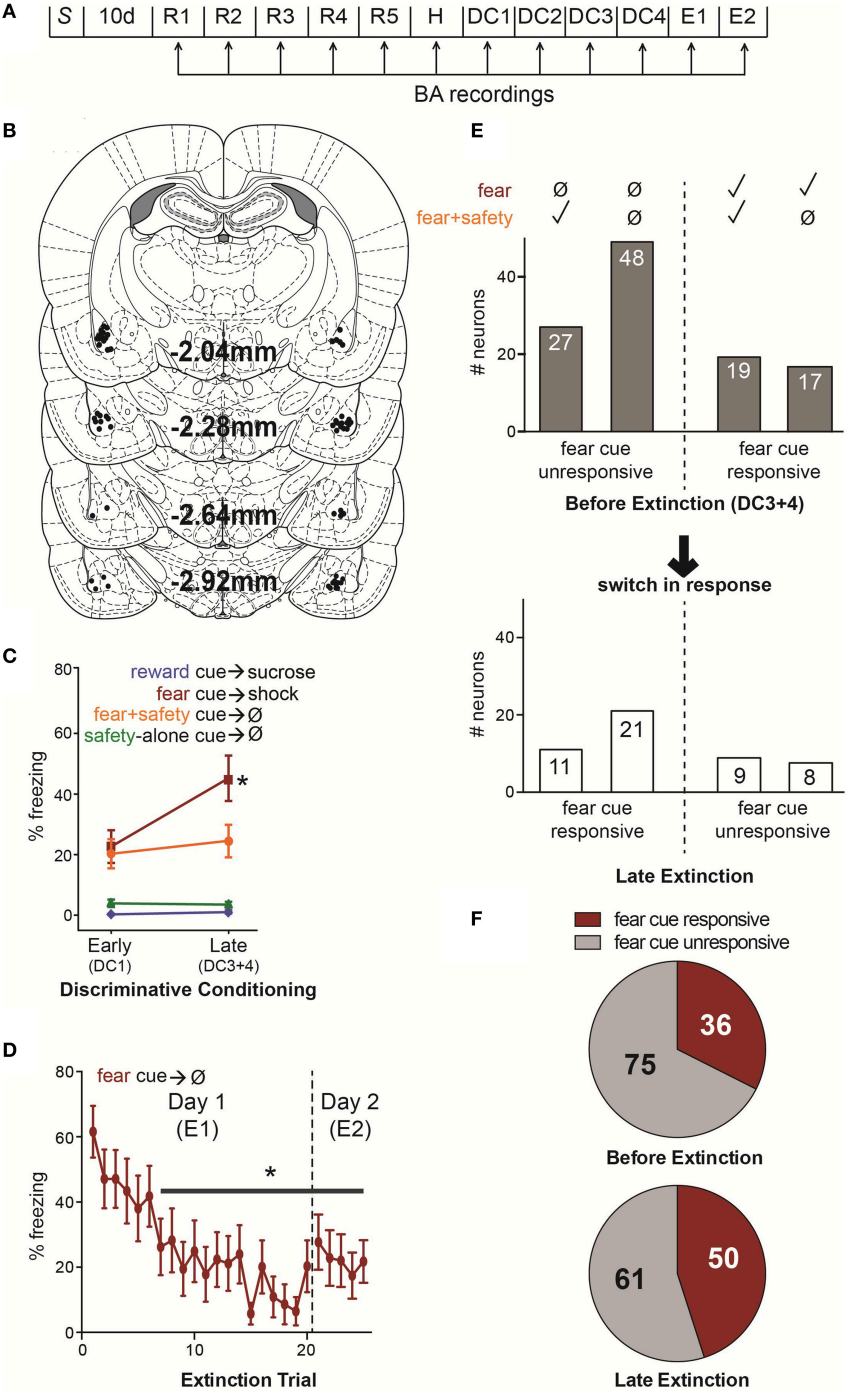
**(A)** Summary of experimental design. S, surgical implantation of electrodes into the BA bilaterally followed by 10 d surgical recovery. R1–5, reward sessions in which the reward cue was paired with sucrose delivery. H, habituation in which, in addition to the reward cue-sucrose pairings, rats also received unreinforced presentations of the future fear and safety cues. DC1–4, discriminative conditioning in which reward cue-sucrose pairings continued as well as the addition of trials where the fear cue was paired with footshock, the fear cue was paired with the safety cue without footshock, or the safety cue was presented alone without footshock. E1–2, extinction in which the fear and reward cues were presented unreinforced. **(B)** Locations of each electrode tip from 14 rats. All 111 recorded neurons were in the BA. **(C)** Mean (±SEM) percentage of time spent freezing during each cue comparing early vs. late DC sessions (DC1 vs. DC3+4). During late DC, rats froze significantly more to the fear cue compared to the fear+safety cue, reward cue or safety-alone cue, demonstrating discriminatory fear behavior (^*^p < 0.05). **(D)** Mean (±SEM) percentage of time spent freezing for each fear cue trial during E1 and E2. Freezing was significantly suppressed compared to the first trial beginning at trial 7 and remained significantly suppressed for the remainder of trials during E1 and E2 (^*^p < 0.05). **(E)** Summary of fear cue unresponsive and responsive neurons before extinction and during late extinction. Above, neurons were assigned to one of four groups based on their response to the fear cue and fear+safety cue during late DC (DC3+4); i.e., before extinction. A neuron was considered responsive if there was a significant change in firing frequency during the first 200 ms of the cue compared to pre-cue baseline. Below, a summary of the subset of neurons from each of the four groups to switch their response to the fear cue during late extinction (trials 10–20 of E1 and trials 1–5 of E2 in which freezing behavior was significantly lowered). From left to right, before extinction, one group (*n* = 27) showed no response to the fear cue but did show a significant change in firing frequency in response to the fear+safety cue. During late extinction, 11 of these neurons switched to being fear cue responsive. The next group (*n* = 48) showed no response to either the fear or fear+safety cue before extinction. But during late extinction, 21 of these neurons became fear cue responsive. In contrast, the next group (*n* = 19) showed a significant change in firing frequency in response to both the fear and fear+safety cue before extinction and nine of these neurons became fear cue unresponsive during late extinction. The last group (*n* = 17) showed a significant change in firing frequency in response to the fear cue but not the fear+safety cue before extinction. Of these neurons, eight became fear cue unresponsive. **(F)** Comparison of the number of neurons that were fear cue responsive, irrespective to its responding to the other cues, before extinction (DC3+4) to late extinction. The number of neurons being fear cue responsive increased from 36 before extinction to 50 during late extinction, a 39% increase.

The three cues signifying reward, fear or safety were a 20 s continuous 3 kHz tone (70 dB), a 20 s pulsing 11 kHz tone (200 ms on, 200 ms off; 70 dB) or a 20 s continuous light (28 V, 100 mA), counterbalanced across subjects, with the caveat that the light cue was reserved for the safety cue in most subjects, 12 out of 14 rats. Training first consisted of five reward sessions (Figure 1A; R1–5), in which a 20 s reward cue was paired with 3 s delivery of a 10% sucrose solution (100 μL) into a port accessible to the rat (3 s sucrose delivery commenced pseudorandomly between 10 and 20 s after reward cue onset for 25 trials, ITI 90–130 s). This was followed by a single session of habituation (H) to the future fear cue and safety cue during a session in which reward cue training continued (25 reward trials, ITI 90–130 s). The future fear cue and safety cue were presented separately five times each for 20 s without reinforcement to allow subjects to habituate to their presentation thereby reducing any baseline freezing to these novel cues. Four sessions of discriminative conditioning followed (DC1–4): reward cue training continued (3 s sucrose delivery commenced 18 s after reward cue onset; 15 trials), along with the additional presentation of the 20 s fear cue followed by a mild 0.5 s footshock at the offset of the fear cue (0.4 mA; four trials). On separate trials this same 20 s fear cue was simultaneously paired with a 20 s safety cue resulting in no footshock (fear+safety cue; 15 trials). Trials in which the 20 s safety cue was presented alone without any footshock were also included (safety-alone cue; 10 trials) to assess if any freezing developed to the safety cue as a result of being paired to the fear cue as well as providing the animal with additional trials that contained a safety cue-no shock contingency. Trials were presented pseudorandomly (ITI 100–140 s). Two sessions of extinction followed (E1–2), in which the fear and reward cues were presented unreinforced (E1: 20 trials each of the fear and reward cues, E2: five trials each of the fear and reward cues; trials were presented pseudorandomly, ITI 90–130 s).

### Behavioral analyses

Fear behavior was assessed, offline from videos, by measuring freezing, defined as complete immobility with the exception of respiratory movements, which is an innate defensive behavior (Blanchard and Blanchard, 1969; Fendt and Fanselow, 1999). The total time spent freezing was quantified during the entire 20 s of each cue presentation and expressed as percent time spent freezing. Calculating the percent time spent in the port assessed reward-seeking behavior. Behavioral data were analyzed using one- or two-way repeated measures ANOVA, followed by Tukey's *post-hoc* test when indicated by significant (*p* < 0.05) main effects or interactions.

### Surgery

Rats were anesthetized with isoflurane and stereotaxically implanted bilaterally with fixed eight-electrode arrays (NeuroBiological Laboratories) directed at the BA (relative to bregma: AP: −2.04 to −2.92 mm posterior, ML: 4.1–4.9 mm, DV: 6.6–7.5 mm ventral from brain surface (Paxinos and Watson, 2007) (Figure 1B). Rats were allowed 7–10 d to recover in which they had *ad libitum* access to food and water.

### *In vivo* single unit recordings

Neuronal activity was recorded with commercial hardware and software, including headstage amplifiers and programmable amplifiers, filters (0.4 and 5 KHz), and multichannel spike-sorting software (Plexon). Implanted rats were connected to the recording apparatus via a swivel commutator. Discrimination of individual units was performed offline by using principal component analysis of waveform shape. Single cells were identified by constancy of waveform shape, cross-correlograms, and interspike intervals (Janak, 2002). In addition, quantitative J3 and Davies Bouldin validity index (DB) statistics were calculated. High J3 values and low DB values are indicative of good single unit isolation (Davies and Bouldin, 1979; Nicolelis et al., 2003; Herry et al., 2008; Sangha et al., 2013). Stability of units across sessions was assessed by calculating principal component space cylinders using WaveTracker (Plexon). In addition, linear correlation values between time-shifted average waveforms were calculated (Jackson and Fetz, 2007; Herry et al., 2008; Sangha et al., 2013). As a control, the *r*-values from average waveforms of randomly paired neurons and sessions were computed. Only units deemed stable across sessions using these procedures were included in the analysis.

### Classification of neurons

For each neuron, significance of cue-evoked firing rates was determined as previously published (Sangha et al., 2013), using a 10,000-round paired permutation test (Hesterberg et al., 2005) comparing the averaged 20 s pre-cue baseline period to the first 200 ms after cue onset during the last two DC sessions and during late extinction (trials 10–20 of E1 and trials 1–5 of E2). That is, the 20 s pre-cue baseline firing rates and the 200 ms post-cue firing rates for a given cue were shuffled and redistributed independently 10,000 times. The differences between the baseline and post-cue firing for the single real case and the 10,000 reshuffled cases were used to create a distribution. In accordance with the permutation test, if the actual mean difference was within < 2.5% of either tail, it was considered significant. *P*-values were then adjusted for multiple corrections using the Benjamini-Hochberg correction with a corrected cutoff of 0.05 (Benjamini and Hochberg, 1995). To avoid false positives, neurons that showed a significant cue-evoked inhibition using this permutation test were only included in the final analyses if the baseline firing frequency was >0.05 Hz. Neurons were classified as “fear cue responsive” if there was a significant increase or decrease in firing rate to the fear cue during late DC. These neurons were then segregated based on whether there was also a significant change in firing rate to the fear+safety cue. Neurons that did not show a significant change in firing rate to the fear cue during late DC (i.e., before extinction) were classified as “fear cue unresponsive.” A subset of these neurons did however show a significant increase or decrease in firing rate compared to baseline to the fear+safety cue and were analyzed separately. Similarly, neurons were classified as “reward cue responsive” if there was a significant increase or decrease in firing rate to the reward cue during late DC.

### Histology

Rats were deeply anesthetized with sodium pentobarbital. A 10 s 19 μA DC current was passed through each wire to mark each electrode tip. Rats were then perfused with formalin containing 3% potassium ferrocyanide. Sections (50 μM) were stained against acetylcholinesterase and only units recorded from electrode wires verified to be in the BA were included in the analyses.

## Results

In a previous study neurons of the BA were tracked over the course of a discriminative conditioning task (Sangha et al., 2013). In this task rats learn to discriminate among fear, safety, and reward cues. In the present study, the same BA neurons were followed into fear and reward cue extinction to assess the plasticity of neurons that were fear cue responsive and fear cue unresponsive before extinction.

Recordings were made during each behavioral training session (Figure 1A, see Materials and Methods). A total of 111 single neurons located in the BA from 14 rats (Figure 1B) were isolated from recordings made during discriminative conditioning and extinction. Most neurons had low mean firing rates (Median = 0.83 Hz, Max = 20.35 Hz, Min = 0.06 Hz), suggesting the sample was predominantly putative projection neurons (Likhtik et al., 2006).

### Fear behavior

#### Discriminative conditioning

The percent time spent freezing during each cue was averaged across early (first DC session, DC1) and late (final two DC sessions, DC3+4) discriminative conditioning (Figure 1C). A Two-way repeated-measures ANOVA on percent time spent freezing revealed a significant interaction between phase of training and cue type [*F*_(3, 39)_ = 8.575, *p* < 0.001] and a main effect of phase of training [*F*_(1, 13)_ = 5.118, *p* < 0.05] and cue type [*F*_(3, 39)_ = 29.331, *p* < 0.001]. Freezing to the fear cue was significantly greater than the fear+safety cues, safety-alone cue, and reward cue during late DC (*post-hoc* Tukey's, *p* < 0.001 each comparison), demonstrating discriminatory fear behavior by these animals.

#### Fear extinction

The percent time spent freezing during each fear cue trial of E1 and E2 was averaged across animals (Figure 1D). A One-way repeated-measures ANOVA on percent time spent freezing revealed a main effect of trial [*F*_(24, 336)_ = 6.35, *p* < 0.0001] and Dunnett's multiple comparisons test showed freezing was significantly lower (*p* < 0.05) during trial 7 and each subsequent trial compared to the first trial. Thus, freezing was significantly suppressed compared to the first trial beginning at trial 7 and remained significantly suppressed for the remainder of trials during E1 and E2.

### Neural recordings of fear and safety neurons

In order to compare neuronal responding during discriminatory fear behavior to significant fear suppression during extinction, neuronal responding was analyzed during late DC (DC3+4) and compared to late extinction (Figure 1E). Late extinction consisted of the last 10 trials of E1 and the 5 trials of E2; freezing behavior during each of these trials was significantly lower than the beginning of extinction (trial 1 of E1, Figure 1D). Z-scores were calculated for each neuron's response to the first 200 ms of each cue (see Materials and Methods) and used to make comparisons among different neuronal populations.

#### Neurons unresponsive to the fear cue before extinction

Neurons classified as “fear cue unresponsive” before extinction had no significant change in firing rates to the fear cue compared to baseline during DC3+4 (permutation tests, *p* > 0.05). These neurons were then segregated based on whether or not they showed significant changes in firing rates to the fear+safety cue compared to baseline during DC3+4 (Figure 1E). This was done in an effort to assess if the “safety” neurons become “extinction” neurons. In other words, does one subpopulation preferentially switch to being fear cue responsive?

**Figure 2:**
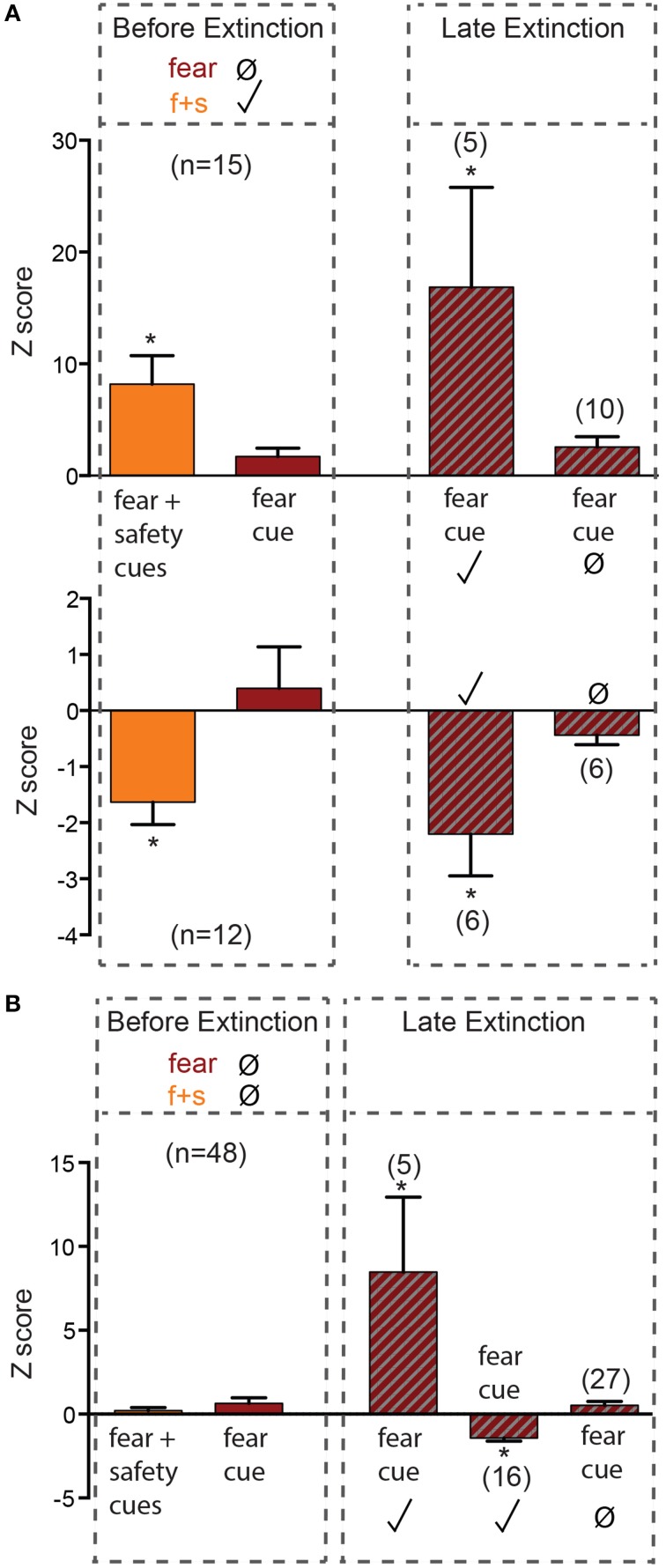
**Neurons unresponsive to the fear cue before extinction**. Z-scores were calculated for each neuron's response to the first 200 ms of each cue. Mean (±SEM) Z-scores are shown to the fear and fear+safety (f+s) cues before extinction and to the fear cue during late extinction. ^*^p < 0.05, firing frequency during first 200 ms of cue of a given neuron compared to its pre-cue baseline firing frequency. Significant positive Z-score values indicate an excitatory response and significant negative Z-score values indicate an inhibitory response. Non-significant values indicate unresponsive to the cue. **(A)** Neurons that were fear cue unresponsive but fear+safety cues responsive before extinction. Five of these fear cue unresponsive neurons developed an excitatory response to the fear cue in late extinction and six developed an inhibitory response. **(B)** Neurons that were both fear cue and fear+safety cue unresponsive before extinction. Of these 48 unresponsive neurons, five developed an excitatory response in late extinction and 16 developed an inhibitory response to the fear cue. The remaining 27 neurons remained unresponsive to the fear cue.

Before extinction, 27 neurons were fear cue unresponsive but fear+safety cue responsive (Figures 1E, 2A), showing a discriminative response to the fear+safety cue vs. fear cue. This subpopulation showed either an excitatory (*n* = 15, Figure 2A upper) or inhibitory (*n* = 12, Figure 2A lower) response to the fear+safety cue. Five of these fear cue unresponsive neurons developed an excitatory response to the fear cue in late extinction and six developed an inhibitory response (permutation tests, *p* < 0.05). The remaining 16 neurons remained unresponsive to the fear cue (permutation tests, *p* > 0.05).

Before extinction, 48 neurons were both fear cue and fear+safety cue unresponsive (Figures 1E, 2B). Of these 48 unresponsive neurons, five developed an excitatory response to the fear cue in late extinction and 16 developed an inhibitory response (permutation tests, *p* < 0.05). The remaining 27 neurons remained unresponsive to the fear cue (permutation tests, *p* > 0.05).

Together, of all the neurons that were unresponsive to the fear cue before extinction (*n* = 76), 43% (32 of 76 neurons) switched to being responsive during late extinction (Figure 1E). Contrary to the hypothesis, neurons that responded to the fear+safety cue, but not the fear cue, before extinction did not appear to preferentially switch to being fear cue responsive during late extinction compared to neurons unresponsive to both cues.

#### Neurons responsive to the fear cue before extinction

Neurons classified as “fear cue responsive” before extinction had significant increases or decreases in firing rates to the fear cue compared to baseline during DC3+4 (permutation tests, *p* < 0.05). These neurons were then segregated based on whether or not they also showed significant changes in firing rates to the fear+safety cues compared to baseline during DC3+4 (Figure 1E). This was done to assess if one subpopulation preferentially switched to being fear cue unresponsive.

**Figure 3:**
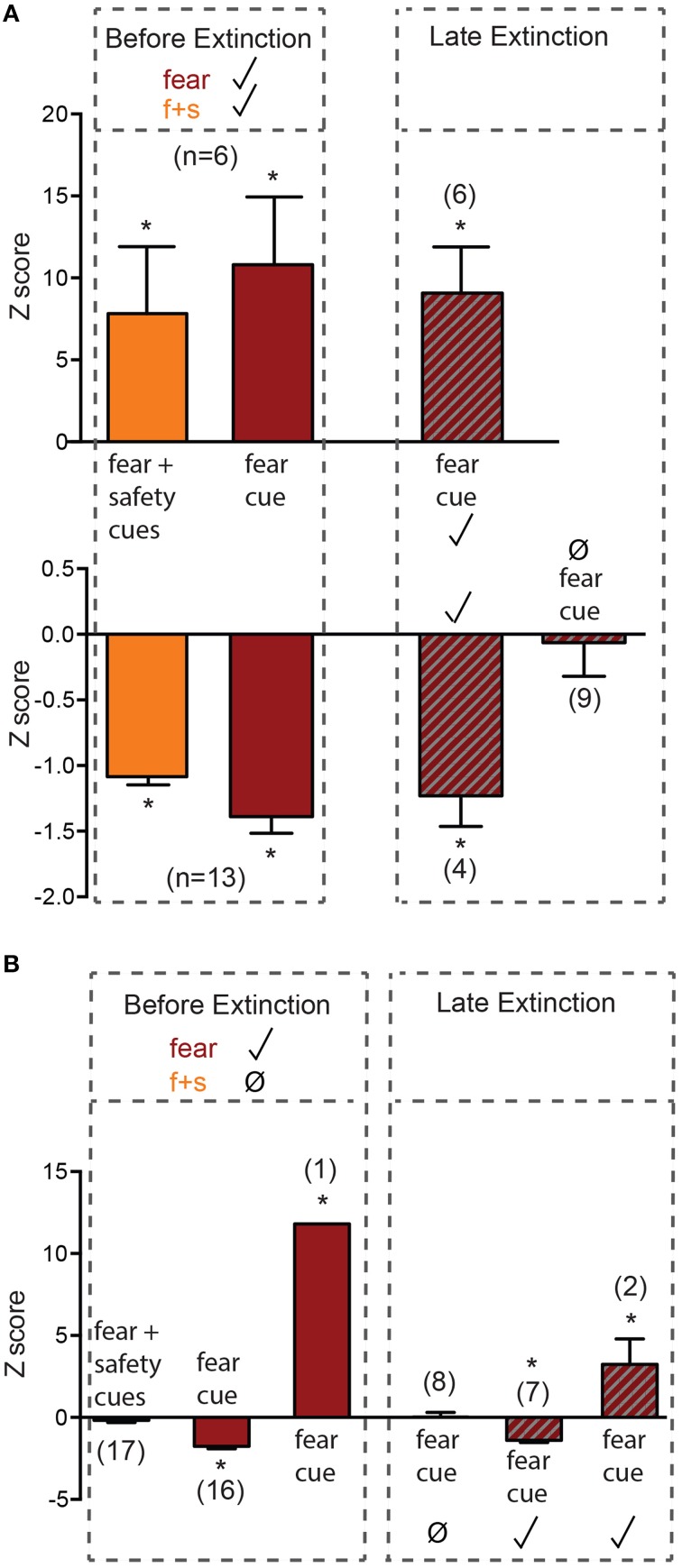
**Neurons responsive to the fear cue before extinction**. Z-scores were calculated for each neuron's response to each cue. Mean (±SEM) Z-scores are shown to the fear and fear+safety (f+s) cues before extinction and to the fear cue during late extinction. ^*^p < 0.05, firing frequency during first 200 ms of cue of a given neuron compared to its pre-cue baseline firing frequency. Significant positive Z-score values indicate an excitatory response and significant negative Z-score values indicate an inhibitory response. Non-significant values indicate unresponsive to the cue. **(A)** Neurons that were both fear cue and fear+safety cues responsive before extinction. All neurons showing an excitatory response to both types of cues before extinction maintained their response through late extinction (*n* = 6). Nine neurons showing an inhibitory response to both cues before extinction lost their inhibitory response in late extinction. The remaining 4 neurons maintained their inhibitory response through late extinction. **(B)** Neurons that were fear cue responsive but fear+safety cue unresponsive before extinction. Eight neurons that showed significant inhibition to the fear cue before extinction lost the inhibitory response in late extinction; one neuron switched its inhibitory response to the fear cue before extinction to an excitatory response to the fear cue in late extinction. The remaining one excitatory response and seven inhibitory response neurons maintained their responses through late extinction.

Before extinction, 19 neurons were both fear cue and fear+safety cue responsive (nondiscriminative; Figures 1E, 3A). This subpopulation showed either an excitatory (*n* = 6, Figure 3A upper) or inhibitory (*n* = 13, Figure 3A lower) response to the fear+safety cue. All neurons showing an excitatory response to both types of cues before extinction maintained their response through late extinction (*n* = 6; permutation tests, *p* < 0.05). Nine neurons showing an inhibitory response to both cues before extinction lost their inhibitory response in late extinction (permutation tests, *p* > 0.05). The remaining four neurons maintained their inhibitory response through late extinction (permutation tests, *p* < 0.05). That is, within this subpopulation of neurons, all excitation responses were maintained through extinction but the majority of inhibition responses were lost through extinction.

Before extinction, 17 neurons were fear cue responsive but fear+safety cue unresponsive (Figures 1E, 3B), showing a discriminative response to the fear cue vs. fear+safety cue. Only 1 neuron showed an excitatory response to the fear cue (Figure 3B) while the remaining 16 neurons showed an inhibitory response to the fear cue. Eight neurons that showed significant inhibition to the fear cue before extinction lost the inhibitory response in late extinction (permutation tests, *p* > 0.05) and one neuron switched its inhibitory response to the fear cue before extinction to an excitatory response to the fear cue in late extinction. The remaining one excitatory response and seven inhibitory responses were maintained through late extinction (permutation tests, *p* < 0.05).

Together, of all the neurons that were responsive to the fear cue before extinction (*n* = 36), 47% (17 of 36 neurons) switched to being unresponsive during late extinction (Figure 1E).

In summary, extinction induced a gain in response to the fear cue in 43% of fear cue unresponsive neurons and a loss in response to the fear cue in 47% of fear cue responsive neurons. The number of fear cue responsive neurons before extinction was also compared to late extinction (Figure 1F) to determine if there was an overall increase or decrease in the absolute number of neurons being fear cue responsive as a result of extinction. Before extinction, 75 neurons were fear cue unresponsive and 36 were fear cue responsive (Figure 1F, upper). During late extinction, 61 neurons were fear cue unresponsive and 50 were fear cue responsive (Figure 1F, lower). Thus, there was a 39% increase in the number of fear cue responsive neurons as a result of extinction. However, a Fisher's exact test revealed this increase was not significant (*p* = 0.073).

### Reward behavior and neural recordings of reward responsive neurons

Since reward cue extinction occurred concurrently to fear cue extinction, neuronal responding to the reward cue before extinction (DC3+4) and during late extinction was also assessed.

#### Discriminative conditioning

**Figure 4:**
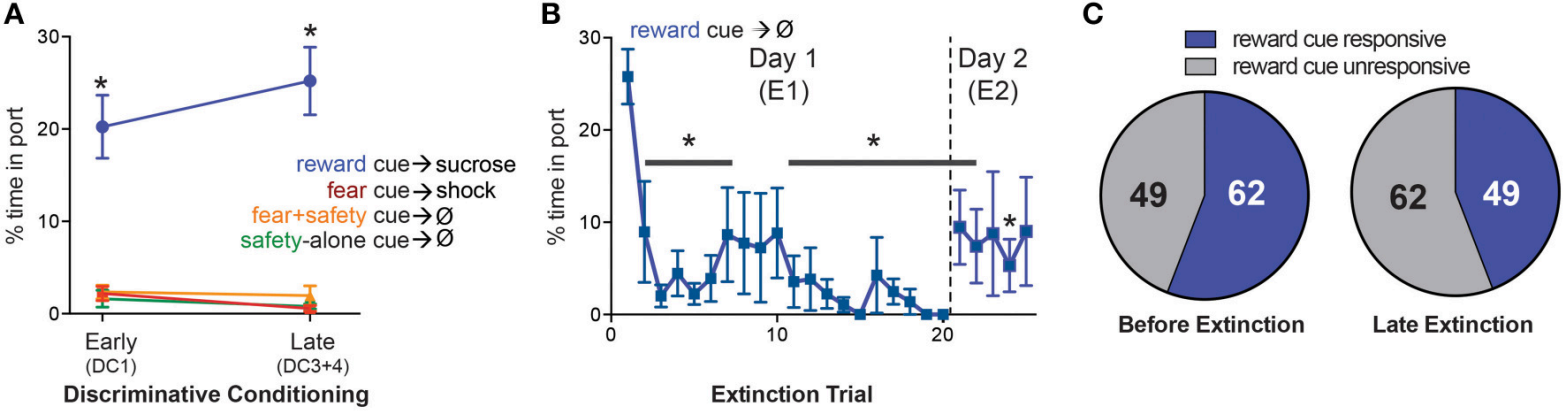
**Changes in response to the reward cue. (A)** Mean (±SEM) percentage of time spent in reward port during each cue comparing early vs. late DC sessions (DC1 vs. DC3+4). During both early and late DC, rats spent significantly more time in the port during the reward cue compared to the fear+safety cue, fear cue or safety-alone cue, demonstrating discriminatory reward-seeking behavior (^*^p < 0.05). **(B)** Mean (±SEM) percentage of time spent in reward port for each reward cue trial during E1 and E2. Reward seeking was significantly suppressed (^*^p < 0.05) compared to the first trial beginning at trial 2 and remained significantly suppressed for the remainder of trials during E1 and E2 with the exception of trials #8–10 of E1 and trials #3 and 5 of E2. **(C)** Comparison of the number of neurons that were reward cue responsive before extinction (DC3+4) to late extinction. The number of neurons being reward cue responsive decreased from 62 before extinction to 49 during late extinction.

The percent time spent in the reward port during each cue was averaged across early (first DC session, DC1) and late (final two DC sessions, DC3+4) discriminative conditioning (Figure 4A). A Two-way repeated-measures ANOVA on percent time spent in port revealed a significant main effect of cue type [*F*_(3, 39)_ = 71.56, *p* < 0.0001]. Reward seeking during the reward cue was significantly greater than the fear+safety cues, safety-alone cue, and fear cue during both early and late DC (*post-hoc* Tukey's, *p* < 0.001 each comparison), demonstrating discriminatory reward seeking behavior by these animals.

#### Reward extinction

The percent time spent in the reward port during each reward cue trial of E1 and E2 was averaged across animals (Figure 4B). A One-way repeated-measures ANOVA on percent time spent in port revealed a main effect of trial [*F*_(24, 336)_ = 2.858, *p* < 0.05] and Dunnett's multiple comparisons test showed reward seeking was significantly lower (*p* < 0.05) during trial 2 and each subsequent trial compared to the first trial with the exception of trials #8, 9, and 10 of E1 and trials #3 and 5 of E2. Thus, compared to the first trial of E1, reward seeking was successfully extinguished by the end of E1 and maintained into E2.

#### Neural recording

Similar to the analyses completed for the fear responsive neurons, the number of reward cue responsive neurons before extinction was compared to late extinction (Figure 4C) to determine if there was an overall increase or decrease in the absolute number of neurons being reward cue responsive as a result of extinction. Before extinction, 62 neurons were reward cue responsive and 49 were reward cue unresponsive. During late extinction, 49 neurons were fear cue responsive and 62 were fear cue unresponsive. This decrease in the number of reward cue responsive neurons as a result of extinction was not significant (Fisher's exact test, *p* > 0.05).

## Discussion

This study examined how neurons classified as discriminative, nondiscriminative or unresponsive during discriminative conditioning (DC), based on their responses to the fear and fear+safety cues, responded to the fear cue during extinction training and recall as fear behavior decreased. The hypothesis tested was that there is a bias for the neurons that were safety cue responsive during DC to become responsive to the fear cue as extinction progresses.

Although 41% of “safety” neurons became fear cue responsive as a result of extinction, the data revealed that there was no bias for these neurons to become preferentially responsive during fear extinction compared to the other identified subgroups. In addition to the plasticity seen in the “safety” neurons, 44% of neurons unresponsive to either the fear cue or fear+safety cue during DC became fear cue responsive during extinction. Together these emergent responses to the fear cue as a result of extinction support the hypothesis that new learning underlies extinction. The overall increase in fear cue responsive neurons in response to extinction also implies that these changes in neuronal responding during extinction are not a result of simple exposure to the sensory stimuli. If the shift were a result of repeated sensory exposures, one would expect the neurons across all groups to show decreased responding to sensory stimuli after multiple exposures as a result of sensory habituation. In contrast, 47% of neurons responsive to the fear cue during DC, regardless of its response to the fear+safety cue, became unresponsive to the fear cue during extinction. These findings are consistent with a suppression of neural responding mediated by inhibitory learning, or, potentially, by direct unlearning. Together, the data support extinction as an active process involving both gains and losses of responses to the fear cue.

The prevalent view in the extinction field is that extinction is an active process, not a passive one (reviewed in Myers and Davis, 2002, 2007). There is ample evidence that extinction does not erase fear memories. In particular, it has been demonstrated by others (Repa et al., 2001; Herry et al., 2008; An et al., 2012), and here in this study (Figure 3A, upper), that amygdala neurons maintain increased responsiveness to the CS, even after extinction. However, there is also evidence that extinction reverses the changes induced by fear learning. For example, fear conditioning-induced potentiation is reversed with extinction in both the thalamo-lateral amygdala and cortico-lateral amygdala pathways (Kim et al., 2007; Hong et al., 2009). The data in the current study are in agreement with both views. There was both a gain of response to the fear cue (Figures 2A,B), which supports extinction as new learning, and a loss of response to the fear cue (Figure 3A, lower and Figure 3B), which may be due to unlearning.

Fear conditioning also induces synchronization at theta frequencies within the amygdala-hippocampal-prefrontal cortex (PFC) network (Sangha et al., 2009; Lesting et al., 2011). After extinction the synchronization between the amygdala and hippocampus is lost but theta synchronization is maintained between the amygdala and PFC, and between the hippocampus and PFC. A similar effect has been reported in the PFC-BA circuit in which fear extinction decreases excitatory transmission from PFC to BA while maintaining inhibitory transmission (Cho et al., 2013). These data demonstrate both reversal and maintenance of learning-induced network activity occurring in parallel during extinction.

This suggests that both new learning and unlearning mechanisms may occur in parallel during extinction. Both processes are active processes. During extinction, a new association regarding the CS is learned; i.e., a CS-no US association. And, similarly to learning the original association, long-term retention of extinction training requires both RNA and protein synthesis across several learning paradigms and species (reviewed in Lattal et al., 2006). But, since extinction also involves reactivation of the original memory, the integrity of the original memory is vulnerable to disruption through reconsolidation mechanisms. When the original CS-US association is reactivated during extinction, it can be updated via reconsolidation mechanisms resulting in a weakening/reversal of the memory. Extinction and reconsolidation have been demonstrated to occur in parallel in the basolateral amygdala complex during reactivation of a fear memory that is no longer reinforced with shock (Duvarci et al., 2006), supporting a view that both new learning and unlearning mechanisms are at play during extinction. This view is also consistent with reports that briefly reactivating a fear memory before employing fear extinction training results in persistent attenuation of fear in both rats (Monfils et al., 2009) and humans (Schiller et al., 2010). In this case, the brief reactivation of the fear memory may induce unlearning via reconsolidation mechanisms and the extinction training results in the learning of a new CS-no US association.

The unlearning phenomena may be caused by reversal of learning-induced changes at the synapse and within the network, or it may be caused by suppression of neural responding mediated by increased inhibition. Several neurons reported here had decreased firing rates in response to the fear cue during extinction. It is not clear what the source of cue-evoked inhibition, nor its downstream effects, might be. However, it has been shown that the balance between excitation and inhibition in the PFC-BA pathway is shifted toward inhibition after extinction (Cho et al., 2013), suggesting that the upstream source for the inhibitions seen in the data presented here may be the PFC. This would be consistent with the requirement of the infralimbic region of the prefrontal cortex to discriminate between the fear and fear+safety cues in this task (Sangha et al., 2014), and to successfully recall fear extinction (Quirk et al., 2000; Laurent and Westbrook, 2009; Chang and Maren, 2010; Fontanez-Nuin et al., 2011; Sierra-Mercado et al., 2011; Santini et al., 2012; Sangha et al., 2014, but see Do Monte et al., 2015).

In summary, the data implicate multiple levels of plasticity in response to fear extinction that most likely interact with multiple microcircuits within the BA. It also indicates that there may be a general remapping of these neuronal microcircuits within the BA in response to extinction. Together it suggests the final output of the integrated BA circuit to influence fear behavior is a balance of excitation and inhibition, and perhaps reversal of learning-induced changes. Further exploration of the intricacies of upregulating or downregulating these BA microcircuits on downstream targets and their effects on fear behavior will lead to greater understanding of the mechanisms contributing to successful fear inhibition which is compromised in individuals suffering from PTSD and similar disorders.

## Author contributions

SS: experimental design, performed research, analyzed data, wrote manuscript.

### Conflict of interest statement

The author declares that the research was conducted in the absence of any commercial or financial relationships that could be construed as a potential conflict of interest. The reviewer Stefan Herlitze and handling Editor Denise Manahan-Vaughan declared their shared affiliation, and the handling Editor states that the process nevertheless met the standards of a fair and objective review.
